# Correlation between Preoperative Serum Levels of Calcium, Phosphate, and Intact Parathyroid Hormone and Radiological Outcomes in Spinal Interbody Fusion among End-Stage Renal Disease Patients

**DOI:** 10.3390/jcm10225447

**Published:** 2021-11-22

**Authors:** Ting-Yu Shih, Yun-Che Wu, Sheng-Chieh Tseng, Kun-Hui Chen, Chien-Chou Pan, Cheng-Hung Lee

**Affiliations:** 1 Department of Orthopaedics, Taichung Veterans General Hospital, Taichung 40705, Taiwan; tingting5813@gmail.com (T.-Y.S.); wcmin@gmail.com (Y.-C.W.); andy770327@gmail.com (S.-C.T.); khc@vghtc.gov.tw (K.-H.C.); pcchou@vghtc.gov.tw (C.-C.P.); 2 Department of Food Science and Technology, Hung Kuang University, Taichung 43302, Taiwan; 3 College of Medicine, National Chung Hsing University, Taichung 40227, Taiwan

**Keywords:** end-stage renal disease, transforaminal lumbar interbody fusion, anterior cervical discectomy and fusion, cage subsidence, implants loosening, adjacent segment disease

## Abstract

Spinal fusion surgery for end-stage renal disease (ESRD) patients is a clinical challenge. This study aimed to investigate whether postoperative radiological outcomes are related to preoperative serum calcium, phosphate, or intact parathyroid hormone (iPTH) levels in patients with ESRD who underwent spinal interbody fusion surgery. This study included 62-consecutive patients with ESRD who underwent anterior cervical discectomy and fusion (ACDF) or transforaminal lumbar interbody fusion (TLIF) surgery for symptomatic spinal disorder. The most recent preoperative serum calcium, phosphate, and iPTH levels were recorded, and the postoperative radiographic outcomes were assessed. A significant correlation was found between the occurrence of cage subsidence and higher blood phosphate, calcium–phosphate product (Ca × P), and iPTH levels in the TLIF group. The occurrence of pedicle screw loosening was related to higher blood phosphate and Ca × P product in the TLIF group. However, no correlation was found between the fusion grades and the serum levels in either the TLIF or ACDF groups. These results indicated that higher preoperative serum phosphate and Ca × P product are risk factors for both cage subsidence and screw loosening in patients with ESRD who underwent TLIF surgery. Higher iPTH levels are also a possible risk factor for cage subsidence.

## 1. Introduction

In Taiwan, there have been an increasing number of patients with end-stage renal disease (ESRD) requiring dialysis in recent years. Taiwan’s dialysis rate is the highest worldwide [1]. However, the advancement of dialysis technology has greatly increased the life expectancy of patients with ESRD [2]. Meanwhile, spine surgeons are seeing an increasing number of patients with degenerative spine diseases and ESRD. Unlike ordinary patients, spine surgery for patients with ESRD can become a substantial challenge because of its complicated comorbidities, such as susceptibility to infection, osteoporosis, and high in-hospital mortality [3,4].

As shown in the Kidney Disease Improving Global Outcomes guidelines in 2017 [5], patients undergoing dialysis treatment should monitor their serum calcium, phosphate, calcium–phosphate (Ca × P) product, and parathyroid hormone to prevent the occurrence of comorbidities, such as cardiovascular disease caused by vascular calcification and renal osteodystrophy, which may cause poor bone quality and poor surgical outcomes [6,7].

To our knowledge, few studies have reported an association between the radiological outcomes and the serum levels among patients with ESRD. Serum calcium, phosphate, calcium–phosphate product, and intact parathyroid hormone (iPTH) levels are known to reflect metabolic bone disease in patients undergoing hemodialysis [5,8]. It may be assumed that patients with poor control of blood calcium, phosphate values, and parathyroid hormone are more likely to develop renal bone disease, which in turn will cause bone quality decline and implant loosening.

In our clinical practice, we also have noticed that dialysis patients with poorly controlled serum calcium and phosphate levels have a high implant failure rate based on radiographic images following spinal fusion surgery. Therefore, this study aimed to retrospectively explore the relationship between the postsurgical and radiological outcomes after spinal surgery in patients with ESRD and the preoperative blood calcium, phosphate, and parathyroid hormone levels.

## 2. Materials and Methods

### 2.1. Study Population

This is a retrospective single-center study of all patients with ESRD who had received interbody fusion for symptomatic spinal disorders from 2005 to 2019 and were enrolled consecutively from Taichung Veterans General Hospital (TVGH). The study protocol was approved by the institutional review board of TVGH (No. CE21363A). The selection criteria for the patients were as follows: (1) all patients were diagnosed with ESRD and had been undergoing dialysis treatment for at least 1 year; (2) all patients had undergone anterior cervical discectomy and fusion (ACDF) surgery or transforaminal lumbar interbody fusion (TLIF) surgery; (3) the patient’s postoperative clinical imaging data and follow-up records were complete, and they were followed up for at least 2 years. The exclusion criteria were as follows: (1) a history of spinal tumors (including metastases), neuromuscular diseases, spinal infection (e.g., osteomyelitis or discitis), and spinal trauma; (2) incomplete postoperative imaging such as full-length lateral spine radiographs; (3) previous spinal surgery; (4) patients who underwent revision spine surgery within 24 months after their first surgery. Disease diagnosis was based on the International Classification of Diseases, Ninth Revision, Clinical Modification (ICD–9–CM). The diagnosis of ESRD, defined by the catastrophic illness card during the study period, was certified by a nephrologist.

### 2.2. Surgical Methods

Two interbody fusion techniques were included in this study: (1) ACDF and (2) TLIF. ACDF was performed using a throat area incision to reach and remove the disc. An interbody cage (Fidji Cervical Cage, Zimmer, IN, USA) with an artificial bone graft (SinboneHT Bone Replacement, Purzer, Taipei, Taiwan) was inserted to fuse together the bones above and below the disc. All patients had a plate attached to the front of the spine with screws into each vertebral bone for additional stability across the disc space. TLIF was performed using a paramedian incision in the prone position. The index disc levels of the lumbar spine were exposed, and unilateral laminectomy with inferior facetectomy was performed. After facetectomy and laminectomy was performed, the ligamentum flavum was excised. The traversing nerve root was identified and adequately mobilized, it was retracted medially and protected by nerve root retractor, and further decompression of the lateral recess and the foramen was performed carefully. The annulus was incised using a long-handled scalpel, and a thorough discectomy was performed using a combination of shavers, pituitary forceps, and curettes. The cartilaginous endplates were denuded completely with curettes and rasps to provide a broad area of cancellous bone for solid bony fusion. The artificial and autologous bone grafts and implant cages (CAPSTONE PEEK Spinal System, Minneapolis, MN, USA) were inserted under sufficient exposure. The transpedicular screws were inserted through the pedicles and into the vertebral body. The alignment of the screws position was then checked under fluoroscopy. The rods were applied onto the transpedicular screws. The bone graft was applied over the posterolateral aspect of the spine [9]. All procedures were performed by four experienced orthopedic spine surgeons in our hospital (C.-H.L., K.-H.C., C.-C.P., and Y.-C.W.). The choice of the manufacturer of the cervical plate (for ACDF surgery) and the pedicle screws (for TLIF surgery) depends on the preference of the surgeon.

### 2.3. Laboratory Profiles and Radiographic Assessment

The most recent (within 4.81 (±1.5) days) preoperative serum calcium, phosphate, and iPTH levels were recorded. All data were abstracted from the electronic medical records of TVGH.

For radiographic outcomes, two experienced orthopedic surgeons (T.-Y.S. and S.-C.T.) reviewed each of the plain films and recorded both the C spine and the L spine fusion status at 6, 12, and 24 months postoperatively. Each fusion level was evaluated separately according to the criteria of the fusion grading system first described by Ito et al. [10]. The bone fusion as seen on the radiographs was classified into four grades as follows: grade 1, complete fusion achieved with the bone bridge formation between the upper and lower vertebral bodies; grade 2, bone bridge not formed, but there was no translucency observed around the cages with thick fusion mass formation; grade 3, fusion not achieved with translucency seen around the cages; grade 4, cage sinking into the vertebral body or bone resorption around cages, which indicates pseudoarthrosis. All surgeons were blinded to the patients’ preoperative serum levels.

**Figure 1:**
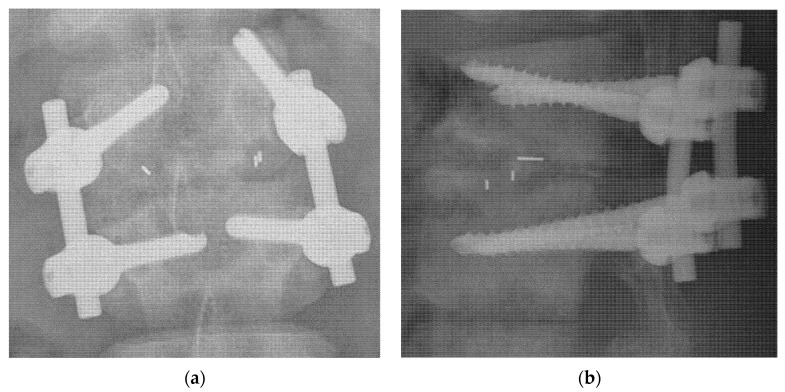
Radiographs after L1–2 TLIF surgery of a 64-year-old woman with ESRD. (**a**) Pedicle screw loosening on the left side of the L1. (**b**) Grade 4 fusion status and grade 3 cage subsidence with bone resorption around cage. TLIF, transforaminal lumbar interbody fusion; ESRD, end-stage renal disease.

From the last postoperative visit, the occurrence of implant loosening, cage subsidence, and adjacent segment disease (ASD) was recorded. X-ray criteria for implant (pedicle screws for TLIF or anterior plate/screw construct for ACDF) loosening were defined as follows: for TLIF surgery, implant loosening was defined as at least a radiolucent zone surrounding the pedicle screw thicker than 1 mm and/or the “double halo” sign [11,12] (Figure 1).

**Figure 2:**
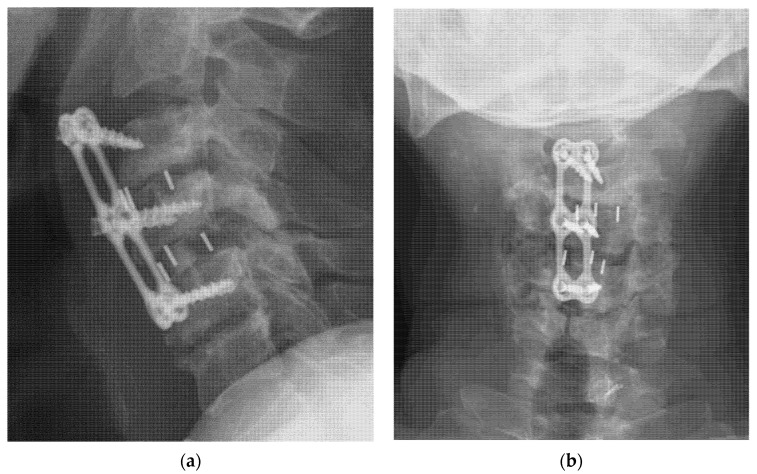
Radiograph after C3–4–5 ACDF surgery of a 50-year-old man with ESRD. (**a**) Lateral view and (**b**) anteroposterior view revealed implant dislocation with grade 4 fusion status at C3–4 and grade 3 at C4–5; grade 0 cage subsidence at both C3–4 and C4–5 levels without progression of neurological symptoms. ACDF, anterior cervical discectomy and fusion; ESRD, end-stage renal disease.

For ACDF surgery, implant loosening was defined as the observation of a ≥2 mm gap between the plate and the anterior aspect of the cervical body compared with the immediate postoperative image findings (Figure 2). Screw loosening was defined when two or more threads were backed out, compared with the postoperative image findings [13]. Subsidence was measured from standing neutral lateral radiographs with parallel endplates at the index level. The degree of the vertebral body collapse around the disc space was categorized according to the grading system first described by Marchi et al.: grade 0, 0–24% collapse; grade I, 25–49% collapse; grade II, 50–74% collapse; and grade III, 75–100% collapse [14,15]. Radiographic ASD in the TLIF group was defined as follows: (1) development of spondylolisthesis of >4 mm, (2) segmental kyphosis of >10°, and (3) adjacent disc collapse [16]. ASD in the ACDF group was defined as follows: (1) the presence of disc space narrowing >25%, (2) new or enlarged osteophytes, (3) anterior/posterior disc herniation, (4) endplate sclerosis, and (5) calcification of the anterior/posterior longitudinal ligaments, as reported in previous studies [17,18].

Intraobserver reliability was assessed by representing the cases to the same examiners, although in different orders, 8 weeks later. Inter- and intraobserver reliabilities were estimated by calculating the kappa coefficient, and the strength of agreement between the examiners was based on Landis and Koch’s classification [19].

### 2.4. Statistical Analyses

Statistical analysis was performed using the Statistical Package for the Social Sciences (SPSS) statistical software version 24 (IBM Corp., Armonk, NY, USA). Kolmogorov–Smirnov test was performed for serum data in each group, and it showed non-normal distribution. Continuous variables were compared between the two groups using the Mann–Whitney U test. Categorical variables were compared using the chi-squared test. The association between the fusion status and the laboratory profiles was assessed using the Spearman’s correlation coefficient. Statistical significance was set at *p* < 0.05.

## 3. Results

### 3.1. Patient Population Demographics

We enrolled 62 patients at 106 levels with ESRD and who received spinal interbody fusion between 2005 and 2019. The demographic data of the patients are presented in Table 1. After surgery, among the 62 patients, two developed pneumonia, one had intestinal obstruction, and seven had poor wound healing and was treated with wound care and oral antibiotics. None had deep infection during follow-up. Among the 62 patients, 36 underwent TLIF surgery, with a total of 53 levels for fusion. A total of 26 patients underwent ACDF surgery, with 53 spinal fusion segments. The mean age of the patients was 65.5 (56.3–73.0) years in the TLIF group and 63.5 (60.8–69.3) years in the ACDF group. There were 19 women and 17 men in the TLIF group and 10 women and 16 men in the ACDF group. The preoperative diagnoses were lumbar spondylolisthesis, lumbar spinal stenosis, and spondylolysis in the TLIF group, and cervical spondylotic radiculopathy and spondylotic myelopathy in the ACDF group. In the comparison between ACDF and TLIF, no significant difference was noted in the demographics between the two groups in terms of sex, age, body mass index, mortality, comorbidity, surgical complication, and the number of fusion segments per patient.

### 3.2. The Relationship between Radiographic Outcomes and Laboratory Data

#### 3.2.1. Interbody Fusion Grade

Regardless of whether the patient with ESRD underwent ACDF or TLIF, no significant relationship was found between the preoperative calcium, phosphate, calcium and phosphate product values, and iPTH and interbody fusion grade at 6, 12, and 24 months after surgery (Table 2).

#### 3.2.2. Cage Subsidence

As shown in Table 3, in the TLIF group, the severity of cage subsidence is significantly correlated with higher blood phosphate values, calcium–phosphate product, and parathyroid hormone (Spearman’s rho coefficient, *p* < 0.05), while in the ACDF group, no such correlation was found.

#### 3.2.3. Implants Loosening

In addition, in the TLIF group, pedicle screw loosening was found to be correlated with higher blood phosphate values and calcium and phosphate products (Mann–Whitney U test, *p* < 0.05). Similarly, no such correlation was found in the ACDF group (Table 4).

#### 3.2.4. ASD

Regardless of the ACDF and TLIF groups, no significant correlation between the occurrence of ASD and preoperative laboratory calcium and phosphate levels, and calcium–phosphate product values. However, interestingly, in the ACDF group, a higher preoperative iPTH level is significantly correlated with a lower occurrence of ASD (Table 4).

The calculated mean kappa coefficient value for the interobserver reliability was 0.91, and the intraobserver reliability after 8 weeks was 0.94. This represents near perfect agreement between the observers and the time points based on Landis and Koch’s measurements [15].

Regardless of whether the patient underwent TLIF or ACDF, most patients with implant loosening/dislocation eventually only received conservative treatments such as painkillers, a back brace, and rehabilitation. Considering the risk of infection caused by reoperation and the poor bone quality of the patients, very few patients undergo revision surgery because of worsening symptoms.

## 4. Discussion

In recent years, the number of patients with ESRD requiring renal replacement therapy has been increasing, and the incidence and prevalence in Taiwan are the highest in the world [1]. Surgery for patients with ESRD is challenging for surgeons. Except for common ESRD-associated comorbidities, complications such as implant loosening, cage subsidence, and ASD were frequently observed in patients who underwent spinal fusion [7,20]. Surgeons should consider perioperative complications such as hardware failure due to poor bone quality and high mortality rates [3].

Han et al. reviewed 12 patients who underwent spinal surgeries among patients with chronic renal failure and reported relatively high complication and mortality rates and low fusion rates [21]. Nyam et al. also reported that patients with ESRD who underwent spinal surgery showed more comorbidities and greater hospital mortality (10.17%) than spinal surgery patients without ESRD (1.39%) [3]. In our study, the overall mortality rate among patients with ESRD was 16%.

In the postoperative radiological outcomes, in a 2014 study by Kanaya et al., of 48 patients with ESRD undergoing lumbar posterolateral fusion with instrumentation, low PTH levels were a risk factor for bone graft failure in patients who had undergone hemodialysis [22]. By contrast, in our study, we did not observe a correlation between iPTH level and interbody fusion rate in either group.

The results of our study show that high serum phosphate, calcium–phosphate product, and iPTH levels in patients with ESRD are risk factors for postoperative cage subsidence in the TLIF group. We also found that a high serum phosphate level and high calcium–phosphate product in the TLIF group were associated with postoperative screw loosening. However, such relationships were not observed in the ACDF group. Although recent evidence reported a risk of loosening of the prosthesis in patients with hip fracture and secondary hyperparathyroidism due to ESRD, we also did not observe a correlation between the iPTH level and the incidence of implant loosening in either group [23]. Surprisingly, a lower iPTH level was associated with the incidence of ASD in the ACDF group.

Hyperphosphatemia is often seen in patients with ESRD due to impaired renal function and the inability to excrete excess phosphate. A high level of serum phosphate is a direct stimulus for vascular calcification, which is one cause of morbid cardiovascular events contributing to the high mortality of chronic kidney disease [24]. Additionally, hyperphosphatemia induces hypocalcemia and hyperparathyroidism, both of which are causes of osteoporosis and poor bone quality in patients with ESRD [8,25,26]. In both our general practice and the previous literature, the incidence of implant loosening may be considerably higher in osteoporotic spines [27,28], and cage subsidence is relevant to low bone mineral density [29,30].

Calcium–phosphate product is a clinically relevant tool for estimating the cardiovascular risk of patients with renal failure. Arterial calcification has been linked to an elevated calcium–phosphate product and increased myocardial calcium content, which is inversely correlated with left ventricular function [6,31]. Elevated calcium–phosphate products are a result of phosphate-induced and secondary hyperparathyroidism-induced bone loss [32,33]. In the TLIF group, our study demonstrated a higher calcium–phosphate product and a higher incidence of implant loosening and cage subsidence, both of which may be related to poor bone quality.

However, in the ACDF group, regardless of the interbody fusion grade incidence of implant loosening, or cage subsidence, we did not observe an association between the serum levels and these radiological outcomes. The only significant finding was that the lower the iPTH level, the higher the possibility of ASD occurrence. In a study on ovariectomized rats, Zhou et al. concluded that PTH has a protective effect on ASD in osteoporotic rats. According to Zhou et al., intermittent administration of PTH prevents ASD. These beneficial actions on adjacent segment discs were accompanied by the maintenance of the integrity and the function of adjacent vertebrae, which is beneficial for the protection of the nutritional pathway of the endplate [34]. Madiraju and colleagues reported that PTH stimulates matrix synthesis and suppresses the markers of calcification potential in degenerated disc cells via the MAPK and PKA signaling pathways [35]. However, in patients with ESRD, secondary hyperparathyroidism may cause metabolic bone disease and extraskeletal calcification [5,8], both of which may induce dismal outcomes. Therefore, in these cases, the results of animal experiments may not be suitable for clinical practice.

This study has several limitations. First, it was a retrospective study with a limited number of patients and operational levels. Second, subjective measurement bias cannot be easily avoided, although there was another experienced physician besides the author participating in the measurement so to alleviate subjective factors. Although the two orthopedists are very experienced in radiographic interpretation, if another neuroradiologist joined in the interpretation as a neutral expert, the results would be more objective. Third, two surgeons who evaluated the radiological outcome were not blinded to the time point. Fourth, dual X-ray absorptiometry (DXA) is the gold standard for measuring osteopenia or osteoporosis, which may cause implant failure in patients with ESRD. However, not every patient in this study had bone density measurements; thus, the bone density value could not be analyzed. The indications for undergoing surgery are not uniform, which will affect the distribution per group. In addition, these patients have been operated on by different surgeons and different implants were used in the procedures. Despite similar technique, this does cause experimental confounding.

When judging interbody fusion, cage subsidence, and adjacent disc degeneration, CT and MRI facilitate judgment more effectively than do X-ray images [36,37]; however, this is not consistent with clinical practice and the national health insurance system protocols in Taiwan. Therefore, very few patients have CT scans or MRI imaging follow-up examinations postoperatively. Our study focused on the postoperative radiological outcomes; however, the postoperative functional evaluations (e.g., EuroQuol 5D and the Oswestry disability Index) are also very important, which were lacking in this article. Implant loosening and cage subsidence are caused by multiple factors. Renal osteodystrophy caused by ESRD is only one of the underlying reasons. According to a previous study, older patients or diabetes patients have higher rates of screw loosening. Secondary osteoporosis is also one of the important factors. However, this study did not analyze these confounding variables [38]. In addition, serum calcium, phosphate values, calcium–phosphate product, and iPTH are not independent variables, in the sense that the blood calcium and phosphate values are likely to affect the calcium–phosphate product and iPTH values. The serum levels during postoperative follow-up were also not presented or compared in this study. Postoperative blood calcium and blood phosphorus levels should also be closely related to the surgical outcomes. Finally, the two-year follow-up may be too short to detect the late loosening of implants.

## 5. Conclusions

According to the above results, patients with ESRD need to strictly control their serum levels before undergoing TLIF surgery, paying special attention to blood phosphate level, calcium–phosphate product, and iPTH level. Higher serum phosphate, iPTH concentration, and calcium–phosphate product values may cause postoperative pedicle screw loosening and cage subsidence. Based on the results of the study, it is recommended to complete the preoperative examination, such as DXA, and treat abnormal blood values and osteoporosis, before performing spinal surgery for patients with ESRD. When performing lumbar spine surgery for ESRD patients, cement augmentation should be considered to avoid short-term implant loosening [39].

## Figures and Tables

**Table 1:** Demographic data and serum levels of the study population stratified by interbody fusion types.

	TLIF (n = 36)		ACDF (n = 26)		*p* Value
**Age (years)**	65.5	±9.2	63.5	±4.3	0.932
**Sex**					0.391
Female	19	(52.8%)	10	(38.5%)	
Male	17	(47.2%)	16	(61.5%)	
**BMI** (kg/m^2^)	23.6	±2.2	22.9	±3.5	0.843
**Mortality**	8	(22.2%)	2	(7.7%)	0.171
**Comorbidity**					
Hyperparathyroidism	2		3		0.641
Peptic ulcer	2		4		0.227
Hypertension	17		16		0.391
CVA	1		0		1.000
CAD	2		4		0.194
DM	12		8		1.000
Atrial fibrillation	2		3		0.641
**Segment(s)**					
1	21	(58.3%)	9	(34.6%)	
2	13	(36.1%)	6	(23.1%)	
3	2	(5.6%)	9	(34.6%)	
5	0	(0%)	1	(3.8%)	
**Serum level**					
Ca (mg/dL)	9.3	(8.3–10.1)	9.8	(8.7–10.4)	0.169
P (mg/dL)	5.3	(3.5–7.2)	4.2	(3.5–5.5)	0.103
Ca × P (mg^2^/dL^2^)	44.6	(29.9–65.5)	42.63	(31.7–49.4)	0.391
iPTH (pg/mL)	206.8	(64.6–714.6)	333	(51.6–760.0)	0.944

*p* Value < 0.05 was consider significant between TLIF and ACDF. Values are expressed as the mean ± standard deviation. TLIF, transforaminal lumbar interbody fusion; ACDF, anterior cervical discectomy and fusion; BMI, body mass index; CVA, cerebrovascular accident; CAD, coronary artery disease; DM, diabetes mellitus; iPTH, intact parathyroid hormone.

**Table 2:** Correlation between fusion grades and serum level of Ca, P, Ca × P, and iPTH in TLIF and ACDF groups.

	Ca	P	Ca × P	iPTH
	**6 months after operation**			
**TLIF**	0.018	0.103	0.099	−0.225
**ACDF**	−0.220	−0.100	−0.153	0.011
	**12 months after operation**			
**TLIF**	0.080	0.130	0.140	0.310
**ACDF**	−0.014	−0.158	−0.106	−0.321
	**24 months after operation**			
**TLIF**	0.091	0.375	0.375	0.384
**ACDF**	0.092	0.126	−0.359	−0.370

Spearman’s rho coefficient. *p* < 0.05, *p* < 0.01; TLIF, transforaminal lumbar interbody fusion; ACDF, anterior cervical discectomy and fusion; iPTH, intact parathyroid hormone; Ca, calcium; P, phosphate; Ca × P, calcium–phospate product; iPTH, intact parathyroid hormone.

**Table 3:** Correlation between grade of cage subsidence and serum level of Ca, P, Ca × P, and iPTH in TLIF and ACDF groups.

Cage Subsidence (Grade)		
	TLIF (*p*-Value)	ACDF (*p*-Value)
**Ca**	0.191 (0.184)	0.123 (0.385)
**P**	0.360 * (0.021)	−0.196 (0.207)
**Ca × P**	0.390 * (0.012)	−0.227 (0.154)
**iPTH**	0.532 ** (0.001)	0.071 (0.680)

Spearman’s rho coefficient. * *p* < 0.05, ** *p* < 0.01. TLIF, transforaminal lumbar interbody fusion; ACDF, anterior cervical discectomy and fusion; iPTH, intact parathyroid hormone; Ca, calcium; P, phosphate; Ca × P, calcium–phospate product; iPTH, intact parathyroid hormone.

**Table 4:** Correlation between the incidence of implants loosening/ASD and serum level of Ca, P, Ca × P, and iPTH in the TLIF and ACDF groups.

	Implant Loosening (+)		Implant Loosening (−)		*p* Value
**TLIF**					
**Ca**	9.1	(8.1–10.0)	9.3	(8.3–10.3)	0.483
**P**	7.2	(3.5–9.0)	4.6	(2.8–5.6)	0.008 **
**Ca × P**	65.57	(35.0–85.5)	38.7	(27.2–54.2)	0.013 *
**iPTH**	389.7	(82.0–1279.0)	121.0	(64.0–274.0)	0.157
**ACDF**					
**Ca**	10.4	(7.9–10.8)	9.2	(8.6–10.0)	0.296
**P**	4.0	(3.3–5.1)	4.9	(3.8–6.0)	0.146
**Ca × P**	42.6	(32.2–46.4)	42.6	(35.0–61.4)	0.484
**iPTH**	708.5	(65.8–855.0)	255.0	(47.1–755.5)	0.054
	**ASD (+)**		**ASD (−)**		***p*** **Value**
**TLIF**					
**Ca**	10.2	(8.9–11.1)	9.2	(8.1–10.0)	0.109
**P**	2.7	(2.5–5.7)	5.6	(3.5–7.2)	0.076
**Ca × P**	30.24	(26.4–50.5)	49.1	(35.0–65.6)	0.163
**iPTH**	68	(44.6–468.0)	208.0	(86.9–916.4)	0.199
**ACDF**					
**Ca**	10.4	(8.7–10.6)	9.5	(8.5–10.4)	0.300
**P**	4.0	(2.9–5.2)	4.3	(3.6–5.7)	0.129
**Ca × P**	32.2	(30.7–50.5)	45.8	(35.0–49.4)	0.124
**iPTH**	60.6	(49.5–318.0)	731.3	(255.0–815.3)	0.002 **

TLIF, transforaminal lumbar interbody fusion; ACDF, anterior cervical discectomy and fusion; iPTH, intact parathyroid hormone; ASD, adjacent segment disease; Ca, calcium; P, phosphate; Ca × P, calcium–phospate product; iPTH, intact parathyroid hormone; * *p* < 0.05, ** *p* < 0.01.

## Data Availability

All data are available upon reasonable request from the corresponding author.
